# Validity and reliability of anxiety literacy (A-Lit) and its relationship with demographic variables in the Iranian general population

**DOI:** 10.3389/fpubh.2024.1359146

**Published:** 2024-04-17

**Authors:** Alireza Jafari, Mahdi Moshki, Ali Mohammad Mokhtari, Fatemehzahra Naddafi, Mahbobeh Nejatian

**Affiliations:** ^1^Department of Health Education and Health Promotion, School of Health, Social Development and Health Promotion Research Center, Gonabad University of Medical Sciences, Gonabad, Iran; ^2^Department of Health Education and Health Promotion, School of Health, Social Determinants of Health Research Center, Gonabad University of Medical Sciences, Gonabad, Iran; ^3^Department of Epidemiology and Biostatistics, School of Health, Social Development and Health Promotion Research Center, Gonabad University of Medical Sciences, Gonabad, Iran; ^4^Student Research Committee, Gonabad University of Medical Sciences, Gonabad, Iran; ^5^Social Determinants of Health Research Center, Gonabad University of Medical Sciences, Gonabad, Iran

**Keywords:** validity, reliability, health literacy, mental health, psychometric

## Abstract

**Introduction:**

Anxiety disorder is one of the most common mental disorders. This cross-sectional research aimed to determine anxiety literacy (A-Lit) psychometric properties among the Iranian population in 2022.

**Methods:**

This research was conducted on 690 people in Iran in 2022. In this study, people were selected by proportional stratified sampling, and the validity and reliability of the A-Lit designed by Griffiths were assessed. Validity of A-Lit was assessed by face validity, content validity, and confirmatory factor analysis. Reliability of A-Lit was evaluated by the McDonald’s omega coefficient, Cronbach’s alpha coefficient, and test– retest. In analytical sections, the tests of One-way ANOVA, Chi-squared test, and independent samples *t*-test were used.

**Results:**

The rates of S-CVI/Ave and CVR for A-Lit were 0.922 and 0.774, respectively. In confirmatory factor analysis, three items were deleted because the factor loading was less than 0.4, and goodness-of-fit indexes (Some of goodness-of-fit indexes: χ2/df = 4.175, GFI: 0.909, RMSEA = 0.068, PCFI = 0.745, AGFI = 0.883) were confirmed as the final model with 19 items. For all items, the Cronbach’s alpha coefficient was 0.832, the McDonald’s omega coefficient was 0.835, and the intraclass correlation coefficient was 0.874. According to the results of this study, 1.3% (*n* = 9) did not answer any questions correctly and 8.4% (*n* = 58) were able to answer 1–6 questions correctly. Approximately 72% (*n* = 495) were able to answer 7–12 questions, and eventually only 18.6% (*n* = 128) were able to answer 13 questions and more. There was a significant relationship between sex, age group, occupation status, marital status, and get information related to mental illness with A-Lit level (*p* < 0.05).

**Conclusion:**

The Persian version of A-Lit was confirmed with 19 items, and this scale is a reliable tool for measuring A-Lit in the general population. The results also showed that a few people have a higher level of anxiety literacy and that educational and intervention programs need to be designed and implemented for the public population.

## Introduction

Anxiety disorder is one of the most common mental disorders (1). Anxiety disorder is characterized by negative feelings and severe arousal, with the main characteristics of fear, doubt, and concern (2). These people often estimate the risk above the actual level and are constantly concerned without the cause (2). Sometimes this concern increases so much that it disrupts one’s daily performance in different areas of life, including job, educational, social, and marital relationships, and may even restrict the individual as a crippling physical illness (2).

Systematic review in 2016 reported that the prevalence of anxiety disorder in the world was between 3.8 and 25% (3). Based on the results of a global study on 204 countries in 2020, before the COVID-19 pandemic, the prevalence of anxiety was 3,824 per 100,000, which was 4,802 per 100,000 after the COVID-19 pandemic, which showed an increase of 25% (4). The findings of a systematic review in the Iranian population showed that 42% had anxiety disorders, 36% in women and 27% in men. In addition, the rate of obvious anxiety was 21% and the hidden anxiety rate was 24% (5).

Anxiety disorder over time can cause other disorders in the body, and there is a relationship between anxiety and physical disorders, and increased anxiety can increase and aggravate these physical disorders (6). Anxiety disorder, if untreated, can be associated with significant personal and social costs due to repeated referrals to receive primary care, reduced productivity in the workplace, unemployment, and increased risk of mood disorders, drug use, and social relationship disorder (7, 8). Severe anxiety also has a strong correlation with depression, and if not considered and no cure for it, the risk of depression increases (9).

Health literacy (HL) is one of the most effective and predictive factors of people’s health (10, 11) and mental health literacy (MHL) is a main strategy in early recognition of mental illnesses and promoting help seeking (12, 13). Anxiety Literacy (A-Lit) is a subsidiary of MHL that examines people’s literacy in the field of anxiety (14). According to the definition of Jorm et al. (15), regarding MHL, A-Lit refers to the set of beliefs and knowledge about anxiety disorders that helps in their recognition, prevention, and management. Therefore, recognizing anxiety disorders and seeking help to prevent and manage them are two key elements of A-Lit.

Whereas, the recognition of social anxiety disorder was low even in developed countries such as the United Kingdom (16). In the Furnham and Lousley (14) study in UK, people’s A-Lit was poor and people were not aware of A-Lit. Also in the Thai Quynh-Chi et al. (16) study in Vietnam, most students had low A-Lit and could not detect anxiety symptoms.

In addition, long delays in seeking mental health services were observed in most people with anxiety disorders. In social anxiety disorder, the interval from the onset of symptoms to seeking treatment is estimated to be between 9 and 28 years and between 6 and 10 years in generalized anxiety disorder (17). As a result, considering the widespread prevalence of anxiety disorders in Iran, measuring A-Lit of society and implementing interventions to promote A-Lit can lead to early diagnosis of anxiety disorders and promotion of help seeking. The prerequisite for implementing these interventions is the existence of a specific, valid, and reliable tool.

In fact, based on the our knowledge, in Iran despite numerous studies that have examined MHL (18, 19), no study has specifically examined A-Lit. The lack of valid and reliable tools may be a possible reason for this problem. After searching and reviewing numerous sources, the only specific tool developed in this regard is (A-Lit, developed by KG) (20). This scale consists of 22 questions that examine the level of literacy in relation to anxiety disorder (20). Due to the lack of an appropriate scale for measuring A-Lit, this research was performed to determine A-Lit psychometric properties among the public population in 2022.

## Methods

This cross-sectional research aimed to determine the psychometric properties of A-Lit among the Iranian population in 2022. Data was collected within 5 months (October 4, 2022 to March 6, 2023).

### Sample size

A sample size of 500 or more is very good for factor analysis (21, 22). In this research, to run the confirmatory factor analysis, a sample size of 800 participants was considered, and finally, a sample size of 690 was analyzed (110 questionnaires were removed in analysis section due to more missing information).

### Sampling method

Proportional stratified sampling was used for selecting participants. First, the number of health centers with their population was determined (*n* = 3). Each health center was considered as a stratum and based on the population of each stratum, the required sample size for each health center was determined. Subsequently, we referred to the health centers and people who had the health record in health centers and had the inclusion criteria were determined. Then, the require samples were selected by simple random sampling method and the questionnaires were given to participants and completed by self-report. People who were unable to completed the questionnaire due to lack of literacy or vision problem, the questionnaire was completed by the interview method and by the questioner. People who were 18 or over the age of 18, having satisfaction and informed consent, had not cognitive problem, and living in Gonabad city were the inclusion criteria.

### Instruments

(1) Demographic part: This section includes characteristics such as age, education level, economic status, sex, occupational status, method of obtaining health information, marital status, and so on.

(2) A-Lit scale: This scale contains 22 questions that examine the A-Lit level. The questions are measured as “False,” “True,” and “I do not know.” Each correct response gets 1 score, and a high score shows high levels of people’s A-Lit. The Cronbach’s alpha and test–test in the study of Gulliver et al. (20) were 0.76 and 0.83.

### Translation and cultural adaptation

At first, permission was obtained from the original designer of the scale. The process of this section was conducted using the World Health Organization Guideline in three parts (23). In the first part, the scale was translated by two experts from English to Persian version and after comparing and reviewing one version of the two Persian versions was created. After that, the Persian version was back translated to the English version and compared with the original English version. In the third part, the back-translated version of the scale was translated to the Persian version, and the final version of the scale was created.

### Validity

Validity of A-Lit was assessed by qualitative face validity, quantitative and qualitative content validity methods, and structural validity. Qualitative face validity of A-Lit was assessed by 9 specialists (experts of Psychology and experts of Health Education and Health Promotion) and 12 participants of target group. Qualitative and quantitative content validity of A-Lit was assessed by 7 specialists (experts of Psychology and experts of Health Education and Health Promotion). For evaluation the quantitative content validity, S-CVI/Ave (scale content validity index averaging) and CVR (content validity ratio) were assessed (24). The value of >0.90 is acceptable for S-CVI/Ave (25). According to the Lawshe table, because the number of evaluators was 7 specialists, the value of above 0.75 is acceptable for CVR (26).

To perform CFA, the software AMOS_V.24_ was used. In this section, the data of outliers were determined and deleted by the Mahalanobis test. Then, data normality was evaluated using kurtosis and skewness tests. To evaluate the model in CFA, the following goodness of fit indexes were used: chi-square ratio to degree of freedom (χ^2^/df), adjusted goodness of fit index (AGFI), root mean square error of approximation (RMSEA), parsimony comparative fit index (PCFI), root mean square residual (RMR), parsimonious normed fit index (PNFI), parsimony goodness of fit index (PGFI), and goodness of fit index (GFI) (27–29). The acceptable goodness of fit indexes is χ^2^/df less than 5, RMR and RMSEA less than 0.08, AGFI more than 0.8, PCFI, PGFI, and PNFI more than 0.5, and GFI more than 0.9 (27–31).

### Reliability

The internal consistency was surveyed using Cronbach’s alpha coefficient using SPSS_V.24_ software. A score ranging from 0.70 to 0.95 is considered acceptable for internal reliability (Cronbach’s alpha) (32, 33). The McDonald’s omega coefficient was evaluated using JASP version 0.11.1. To evaluate the test–retest reliability, in this study, 30 participants were selected and examined twice with a period of 1 month. For test–retest reliability, the intraclass correlation coefficient (ICC) was checked (ICC > 0.80 is acceptable) (34).

### Statistical analysis in analytical sections

In this study the tests of One-way ANOVA, Chi-square, and independent samples *t*-test were used to check the relationship between the qualitative variables with A-Lit by SPSS version 24. Also, in the one-way ANOVA test, post hoc test (Tukey’s honestly significant difference) was used to evaluate the relationships between variables.

## Results

### Demographic characteristics

In this study, most participants were female (*n* = 370, 53.6%), in the age group 18 to 30 (*n* = 354, 51.3%), university students (*n* = 274, 39.7%), had an academic education level (*n* = 463, 67.1%), and medium economic status (*n* = 474, 68.7%). The most common ways to obtain health information were the Internet (*n* = 351, 50.9%) and physician/health care providers (*n* = 148, 21.4%). The most common way to get information related to mental illness was the Internet (*n* = 204, 29.6%) (Table 1).

**Table 1:** Frequency distribution of demographic variables and its relationship with A-Lit (*n* = 690).

Variables		*n* (%)	A-Lit status *n* (%)				*p*-value^c^	A-Lit	*P*-value
			Incorrect response/do not know	Correct response to 1–6 questions	Correct response to 7–12 questions	Correct response to 13 questions and more		Mean (SD)	
Sex	Male	320(46.4)	3(0.9)	41(12.8)	241(75.3)	35(11)	0.010	41.64(4.19)	<0.001^a^
	Female	370(53.6)	6(1.6)	17(4.6)	254(68.6)	93(25.2)		43.23(4.03)	
Age group	18–30	354(51.3)	1 (0.3)	18(5.1)	259(73.2)	76(21.5)	<0.001	43.21(3.88)	<0.001^b^
	31–43	182(26.4)	6(3.3)	22(12.1)	123(67.6)	31(17)		42.17(4.38)	
	44 and more	154(22.3)	2(1.3)	18(11.7)	113(73.4)	21(13.6)		41.22(4.29)	
Occupation status	Housewife	64(9.3)	4(6.3)	5(7.8)	40(62.5)	15(23.4)	0.003	42.17(4.35)	<0.001^b^
	University student	274(39.7)	1(0.4)	13(4.7)	201(73.4)	59(21.5)		43.40(3.84)	
	Employed	202(29.3)	3(1.5)	23(11.4)	135(66.8)	41(20.3)		42.30(4.47)	
	Retired	35(5.1)	0	5(14.3)	27(77.1)	3(8.6)		40.80(4.00)	
	Self-employed	90(13)	1(1.1)	9(10)	71(78.9)	9(10)		41.50(3.94)	
	Laborer	15(2.2)	0	1(6.7)	14(93.3)	0		41.46(3.97)	
	Unemployed	10(1.4)	0	2(20)	7(70)	1(10)		40.80(4.00)	
Marital status	Married	386(55.9)	4(1)	42(10.9)	269(69.7)	71(18.4)	0.060	42.10(4.29)	0.005^a^
	Single	304(44.1)	5(1.6)	16(5.3)	226(74.3)	57(18.8)		43.00(4.00)	
Place of residence	Village	54(7.8)	1(1.9)	2(3.7)	39(72.2)	12(22.2)	0.551	42.70(4.40)	0.706^a^
	City	636(92.2)	8(1.3)	56(8.8)	456(71.7)	116(18.2)		42.47(4.17)	
Education level	Illiteracy	1(0.1)	0	0	1(100)	0	0.010	39(0)	0.126^b^
	Elementary	11(1.6)	1(9.1)	0	7(63.6)	3(27.3)		44.36(4.10)	
	Middle school	20(2.9)	0	4(20)	14(70)	2(10)		40(3.91)	
	High school	195(28.3)	3(1.5)	6(3.1)	158(81)	28(14.4)		42.20(3.97)	
	Academic	463(67.1)	5(1.1)	48(10.4)	315(68)	95(20.5)		42.65(4.27)	
Economic status	Good	149(21.6)	0	12(8.1)	108(72.5)	29(19.5)	0.035	42.36(4.32)	0.290^b^
	Medium	474(68.7)	8(1.7)	37(7.8)	333(70.3)	96(20.3)		42.63(4.22)	
	Weak	67(9.7)	1(1.5)	9(13.4)	54(80.6)	3(4.5)		41.80(3.53)	
Method of obtaining health information	Physician/health care providers	148(21.4)	1(0.7)	4(2.7)	115(77.7)	28(18.9)	<0.001	42.26(4.61)	0.001^b^
	Internet	351(50.9)	2(0.6)	23(6.6)	251(71.5)	75(21.4)		42.99(4.12)	
	Newspapers/magazines	15(2.2)	0	1(6.7)	13(86.7)	1(6.7)		42.40(3.97)	
	Friends and acquaintances	48(7)	1(2.1)	10(20.8)	34(70.8)	3(6.3)		41.35(3.65)	
	Book	47(6.8)	0	4(8.5)	33(70.2)	10(21.3)		43.23(3.29)	
	Radio, television and satellite	61(8.8)	4(6.6)	15(24.6)	33(54.1)	9(14.8)		40.70(3.98)	
	I do not know	19(2.8)	1(5.3)	1(5.3)	16(84.2)	1(5.3)		41.89(4.09)	
Get information related to mental illness	Yes	533(77.2)	5(0.9)	18(3.4)	386(72.4)	124(23.3)	<0.001	43.03(4.28)	<0.001^a^
	No	157(22.8)	4(2.5)	40(25.5)	109(69.4)	4(2.5)		40.66(3.24)	
Method of obtaining information related to mental illness	Physician/health care providers	49(7.1)	1(2)	4(8.2)	35(71.4)	9(18.4)	<0.001	41.18(4.40)	0.047^b^
	Psychologist/psychiatrist	47(6.8)	0	1(2.1)	37(78.7)	9(19.1)		42.53(4.23)	
	Friends and acquaintances	29(4.2)	0	1(3.4)	25(86.2)	3(10.3)		42.75(3.18)	
	Book	60(8.7)	0	3(5)	43(71.7)	14(23.3)		43.66(4.34)	
	Internet	204(29.6)	1(0.5)	6(2.9)	150(73.5)	47(23)		43.19(4.12)	
	Radio, television and satellite	27(3.9)	3(11.1)	0	17(63)	7(25.9)		42.62(4.34)	
	All Items above	132(19.1)	0	7(5.3)	90(68.2)	35(26.5)		43.38(4.47)	

^a^Independent samples *t-*test, ^b^One-way ANOVA, ^c^Chi-square.

### Psychometric section results

#### Validity assessment

In the face validity and content validity, four items were modified and the rates of S-CVI/Ave and CVR for A-Lit were 0.922 and 0.774, respectively.

#### CFA

**Figure 1:**
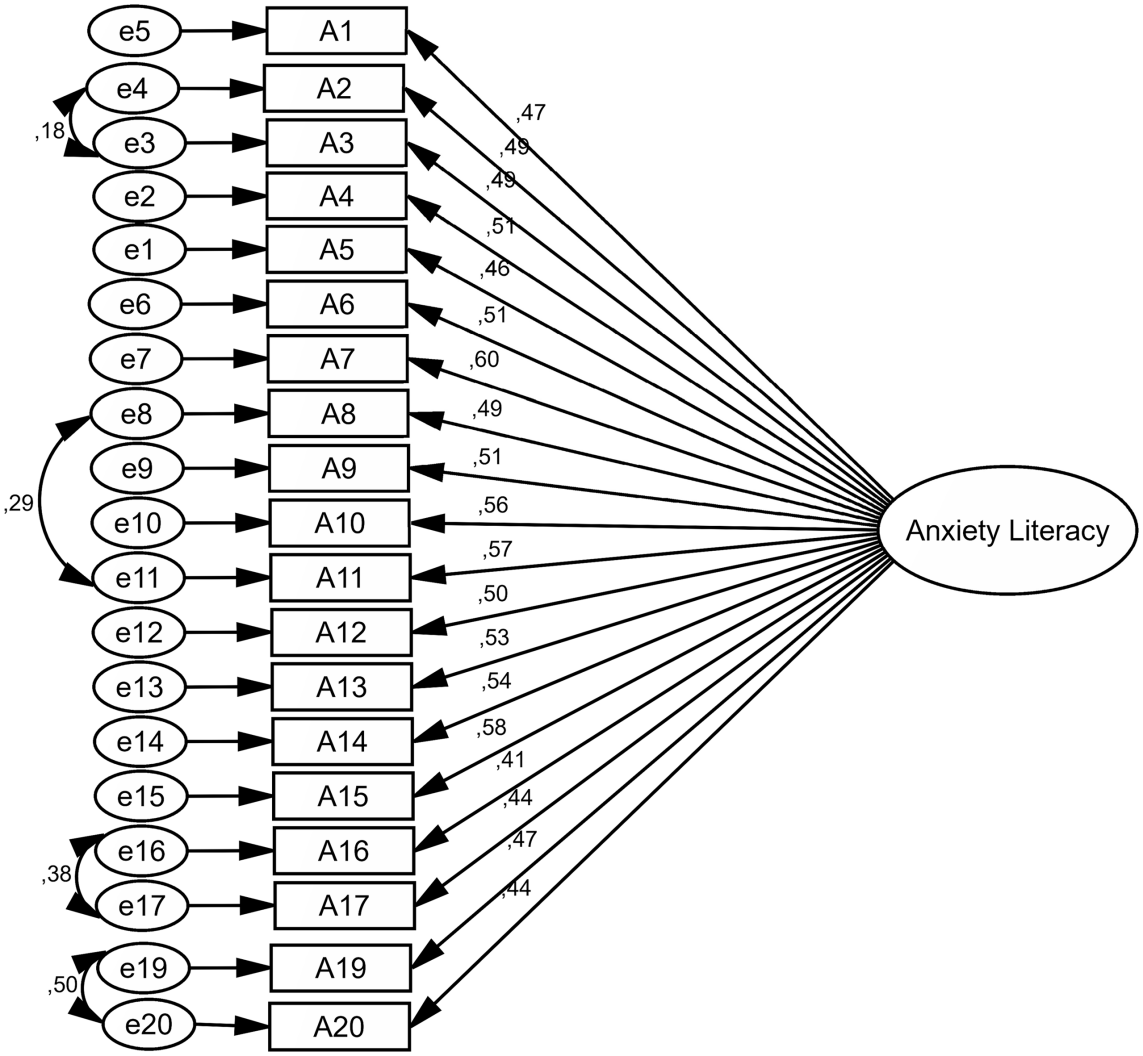
Standardized parameter estimates for the factor structure of the A-Lit scale (all items loadings are significant at *p* < 0.001).

In this section, the goodness of fit indices confirmed the final model (Table 2). The factor loading of all items was above 0.4, and only three questions were deleted because the factor loading was less than 0.4 (Table 3 and Figure 1).

**Table 2:** The model fit indicators of the A-Lit scale.

Goodness of fit indices	CFA	Acceptable value
χ^2^	617.969	–
df	148	–
χ^2^/df	4.175	<5
*p*-value	0.000	>0.05
RMSEA	0.068	<0.08
RMR	0.027	<0.08
AGFI	0.883	>0.8
GFI	0.909	>0.9
PNFI	0.715	>0.5
PGFI	0.708	>0.5
PCFI	0.745	>0.5

**Table 3:** Factor loadings of the A-Lit scale in the CFA among general population.

Items	Factor loadings (standardized regression weights)
1. People with anxiety disorder often speak in a rambling and disjointed way. (False)	0.475
2. Being easily fatigued may be a symptom of anxiety disorder. (True)	0.489
3. Reckless and foolhardy behavior is a common sign of anxiety disorder. (False)	0.494
4. Irritability may be a symptom of anxiety disorder. (True)	0.506
5. Bearing grudges and refusing to forgive others may be a sign of anxiety disorder. (False)	0.465
6. People with anxiety disorder often hear voices that are not there. (False)	0.514
7. Too much worry is the main symptom of anxiety disorder. (True)	0.595
8. Tense muscles may be a symptom of anxiety disorder. (True)	0.487
9. Anxiety disorder does not affect your concentration. (False)	0.507
10. Having several distinct personalities may be a sign of anxiety disorder. (False)	*0.557*
11. A dry mouth can be a symptom of anxiety disorder. (True)	0.570
12. The best way of dealing with anxiety disorder is to handle it yourself. (False)	0.500
13. Generalized anxiety disorder is a common cause of workplace disability. (True)	0.535
14. Generalized anxiety disorder does not run in families. (False)	0.537
15. Being bullied or victimized increases your risk of developing an anxiety disorder. (True)	0.580
16. Antidepressants are effective treatment for anxiety disorder. (True)	0.407
17. Many treatments for anxiety disorder are more effective than antidepressants. (False)	0.445
*18. Acupuncture is just as effective as cognitive behavioral therapy for anxiety disorder. (False)*	Deleted^*^
19. Reading self-help books about cognitive behavioral therapy is not effective for anxiety disorder. (False)	0.470
20. It’s not a problem to stop taking antidepressants quickly. (False)	0.445
*21. Antidepressants are addictive. (False)*	Deleted^*^
*22. Antidepressant medications usually work straight away. (False)*	Deleted^*^

^*^This question was deleted in confirmatory factor analysis stage, because their factor loading value were less than 0.4.

#### Reliability assessment

Cronbach’s alpha was 0.832 for A-Lit. McDonald’s omega coefficient of A-Lit was 0.835. In the test–retest, the rate of intraclass correlation coefficient of A-Lit was 0.874 (95% Confidence Interval, lower bound = 0.751, upper bound = 0.937, *p* < 0.001).

### Descriptive and analytical sections of the results

There was a significant relationship between sex and A-Lit, and the level of A-Lit was higher among women than men (*p* < 0.001). Also, compared to men, more percentage of women were able to answer 13 questions and more (*p* = 0.010). There was a significant relationship between the age group and the A-Lit level, and the age group of 18–30 years gained a higher score on A-Lit (*p* < 0.001). There was a significant statistical relationship between job status and HL, and university students had a higher A-Lit level (*p* < 0.001). Based on the Tukey’s *post hoc*, the age group of 18–30 had more A-Lit level than other groups (*p* < 0.05) (Supplementary Table S1). Other results are visible in Supplementary Table S1.

There was no significant relationship between education level and A-Lit (*p* = 0.126), but those with academic education levels were able to answer 13 questions and more compared to other people (*p* = 0.010). The results also showed that people who obtained general health information from books and the Internet had higher A-Lit scores (*p* = 0.001). People who reported information about mental disorders had a higher level of A-Lit (*p* < 0.001). People who obtained information about mental disorders from the book had higher A-Lit scores (*p* = 0.047). Other information can be found in Table 1.

In this study, 1.3% (*n* = 9) of participants did not answer any questions correctly 8.4% (*n* = 58) were able to answer 1–6 questions correctly. Approximately 72% (*n* = 495) were able to answer 7–12 questions, and eventually only 18.6% (*n* = 128) were able to answer 13 questions and more. The results in Table 4 are related to the accountability status of the participants for each question.

**Table 4:** Participants’ response to the A-Lit scale.

Items	*n* (%)		
	Correct response	Incorrect response	Do not know
1. People with anxiety disorder often speak in a rambling and disjointed way. (False)	354(51.3)	230(33.3)	106(15.4)
2. Being easily fatigued may be a symptom of anxiety disorder. (True)	398(57.7)	196(28.4)	96(13.9)
3. Reckless and foolhardy behavior is a common sign of anxiety disorder. (False)	194(28.1)	387(56.1)	109(15.8)
4. Irritability may be a symptom of anxiety disorder. (True)	420(60.9)	184(26.7)	86(12.5)
5. Bearing grudges and refusing to forgive others may be a sign of anxiety disorder. (False)	332(18.1)	209(30.3)	149(21.6)
6. People with anxiety disorder often hear voices that are not there. (False)	299(43.3)	193(28)	198(28.7)
7. Too much worry is the main symptom of anxiety disorder. (True)	511(74.1)	119(17.2)	60(8.7)
8. Tense muscles may be a symptom of anxiety disorder. (True)	378(54.8)	184(26.7)	128(18.6)
9. Anxiety disorder does not affect your concentration. (False)	499(72.3)	128(18.6)	63(9.1)
10. Having several distinct personalities may be a sign of anxiety disorder. (False)	209(30.3)	305(44.2)	176(25.5)
11. A dry mouth can be a symptom of anxiety disorder. (True)	467(67.7)	111(16.1)	112(16.2)
12. The best way of dealing with anxiety disorder is to handle it yourself. (False)	197(28.6)	397(57.5)	96(13.9)
13. Generalized anxiety disorder is a common cause of workplace disability. (True)	335(48.6)	213(30.9)	142(20.6)
14. Generalized anxiety disorder does not run in families. (False)	426(61.7)	122(17.7)	142(20.6)
15. Being bullied or victimized increases your risk of developing an anxiety disorder. (True)	497(72)	123(17.8)	70(10.1)
16. Antidepressants are effective treatment for anxiety disorder. (True)	226(32.8)	257(37.2)	207(30)
17. Many treatments for anxiety disorder are more effective than antidepressants. (False)	125(18.1)	285(41.3)	280(40.6)
18. Reading self-help books about cognitive behavioral therapy is not effective for anxiety disorder. (False)	472(68.4)	85(12.3)	133 (19.3)
19. It’s not a problem to stop taking antidepressants quickly. (False)	538(78)	46(6.7)	106(15.4)

## Discussion

### Psychometric section

Because the psychometric characteristics of A-Lit have not been examined in Iran, the validity and reliability of the Persian version of the A-Lit scale in the Iranian general population was examined. The original version of the A-Lit scale contains 22 items, and a high score indicates a high level of A-Lit. After evaluating the psychometric properties of A-Lit, 3 questions were removed from the Persian version of the questionnaire because of a factor loading of less than 0.4, and 19 items were confirmed for the modified version.

According to the CFA test, factor loading values were calculated and all questions were larger than 0.4, except for three items. Therefore, at this stage, only three questions were deleted and the final model was confirmed with 19 questions. The cultural and social differences of Iranian society could have been the reason for the elimination of these questions. For example, one of the questions deleted from the Persian version was about acupuncture, which seems to be not well known in the Iranian population. Based on the literature review, there were no studies on the psychometric properties of the A-Lit scale in Iran.

Another study examined the psychometric characteristics of the mental health literacy questionnaire among Iranian soldiers (35). The study showed that two of the 33 cases that entered the content analysis phase were eliminated by a factor loading below 0.35, and the questionnaire was confirmed with 31 items. The results of the psychometric properties of depression literacy (D-Lit) in the Iranian community showed that only one question was eliminated in the confirmatory factor analysis stage, and D-Lit was confirmed with 21 questions (36).

In the reliability stage of the A-lit scale, the Cronbach’s alpha coefficient was 0.832 and McDonald’s omega coefficient was 0.835. In addition, the test–retest results indicate the appropriateness of the intraclass correlation coefficient (ICC = 0.874), which agrees with previous studies. In a study of young Australian athletes, the Cronbach’s alpha coefficient of the A-Lit scale was calculated 0.76, which was acceptable (20). The reliability of the questionnaire was also evaluated by the test–retest, with an acceptable value of 0.83 (20). Another study in Australia on adults with symptoms of untreated social anxiety disorder found that the results of the test–retest for the social A-Lit in the control group was 0.85 (37).

As can be seen, the Persian version of the questionnaire is similar to the original version. Therefore, this scale can be used to measure the level of A-Lit of Iranian population, and if A-Lit is low, an intervention program can be implemented in this regard. Educational interventions can be a way to increase A-Lit and improve help seeking attitudes (38). A clinical trial study showed that if people’s A-Lit is low, intervention to increase social A-lit can be helpful (37). Therefore, the first step in determining the status of A-Lit in any population is the existence of a valid and reliable tool to plan and implement subsequent preventive programs.

### Descriptive and analytical sections

According to the findings of this study, there was a significant relationship between A-Lit and sex, and A-Lit was higher in women than in men. In addition, the percentage of answering to 13 questions and more in women was significantly higher than that in men. There was also a significant statistical relationship about job status, and students had higher A-Lit levels than others. The findings of this study are very similar to those of other studies. A study of Irish teenagers with anxiety disorders showed that girls had better A-Lit than boys (39). Another study of Singapore students 18 to 24 years old and through social media platforms (40) showed that the A-Lit level was significantly higher in female students, with no difference from the present study. In addition, students in medical sciences had higher A-Lit levels than others. In another study conducted in Turkey on medical students showed that girls had higher mental health literacy (41).

In the study of age groups, it was found that the A-Lit level was significantly higher in the age group of 18–30 years than in the others, and they gained a higher score in A-Lit. Because the prevalence of anxiety disorders is usually more from late adolescence, the present study is somewhat justified (42).

There was no significant relationship between the level of education and A-Lit in the present study; however, the percentage of answers to 13 questions and more was higher in academic education than in other people. Although no similar study was found in this field, another study conducted in Turkey and medical students showed that with the increase in students’ academic activities, the level of mental health literacy also increased (41).

In this study, the most important source of health information and the main source of information about mental disorders was Internet. The results of this study showed that people who obtained their health information and their information related to mental illness from books had higher A-Lit scores. In another study on medical students in Turkey, the Internet social media was the main source of health information (41). The results of a study on Iranian students showed that the Internet and health care providers were the main sources of mental health literacy information and that health care providers were the most reliable source of information (43). The results of our study were in line with the results of the Mahmoodi et al. (43) study, and the Internet was the most important source of information. However, there is a difference in the second source of health information that can be due to the differences in the groups being studied because the present study was performed on the general population, whereas the Mahmoodi et al. (43) study was only performed on university students.

While most of the studies found in this field have been conducted in adolescents and young people, in the present study in order to reduce possible sources of bias, We tried to select the participants as much as possible from different population groups. For example, the age difference between the participants and the target population can reduce the generalizability of the study results. Therefore, in the current study, individuals with different ages were examined to increase the generalizability of the results.

Anxiety disorder can be affected by various factors and affects the different functions of individuals. Few people with this disorder are looking for help and receiving help, and they usually have little information about the disorder (37). Therefore, knowing the status of A-Lit and its related factors can help design more effective preventive programs. Also, since anxiety disorder can cause other problems such as physical disorders and as a result impose subsequent costs, with timely identification and treatment of these disorders in the clinical setting, it is possible to prevent the mentioned problems or reduce their severity.

### The application of this study in clinical settings

Cardiovascular disease is the main cause of death in the world and Iran (44, 45). Anxiety is one of the most common problems in patients with cardiovascular disease, including coronary artery disease and heart failure. Anxiety causes coronary artery disease and consequently causes adverse effects and mortality in these patients (46). On the other hand, anxiety disorder is related with weaker glycemic control in diabetic patients, increased night and morning hypertension in patients with hypertension, increased risk of stroke and heart attack (47–50). The existence of A-Lit tools can examine the status of A-Lit in these patients, and based on their condition, appropriate intervention programs can be designed to increase A-Lit, improve help seeking and treatment, improve and implement anxiety management. Therefore, this tool can be used in various studies and among general population and population of patients used by both researchers and health care providers.

### Strengths and limitations

One of the limitations of this study was the lack of similar studies to compare the results. The strengths of this study include the high volume of sample size, conducting the study in the general population, and determination of the status of A-Lit in different groups. While most of the studies found in this field have been conducted in adolescents and young people, in the present study in order to reduce possible sources of bias, We tried to select the participants as much as possible from different population groups. For example, the age difference between the participants and the target population can reduce the generalizability of the study results. Therefore, in the current study, individuals with different ages were examined to increase the generalizability of the results.

## Conclusion

The Persian version of A-Lit was confirmed with 19 items, and this scale is a reliable tool for measuring A-Lit in the general population of Iranian society. The results also showed that a few people have a higher level of A-Lit and that educational and intervention programs need to be designed and implemented for populations with insufficient A-Lit.

## Data availability statement

The original contributions presented in the study are included in the article/Supplementary material, further inquiries can be directed to the corresponding author.

## Ethics statement

The studies involving humans were approved by Ethics Committee of Gonabad University of Medical Sciences (code of ethics: IR.GMU.REC.1401.070). The studies were conducted in accordance with the local legislation and institutional requirements. The participants provided their written informed consent to participate in this study.

## Author contributions

AJ: Conceptualization, Data curation, Formal analysis, Funding acquisition, Investigation, Methodology, Project administration, Resources, Software, Supervision, Validation, Visualization, Writing – original draft, Writing – review & editing. MM: Conceptualization, Formal analysis, Investigation, Methodology, Software, Supervision, Validation, Writing – original draft, Writing – review & editing. AM: Conceptualization, Formal analysis, Investigation, Methodology, Software, Validation, Writing – original draft, Writing – review & editing. FN: Conceptualization, Investigation, Writing – original draft, Writing – review & editing. MN: Conceptualization, Investigation, Methodology, Project administration, Resources, Supervision, Visualization, Writing – original draft, Writing – review & editing.
