# Supplementary material for: Validity and reliability of anxiety literacy (A-Lit) and its relationship with demographic variables in the Iranian general population
Source: Front Public Health. 2024 Apr 17;12:1359146. doi: 10.3389/fpubh.2024.1359146 (PMC11061481; doi:10.3389/fpubh.2024.1359146)
Supplement: Supplementary file 1 [file Table_1.DOCX]

**Table S1**: Results of Tukey's post hoc

| **Variables** | | | **Std. Error** | **95% Confidence Interval** | | **Sig.** |
| --- | --- | --- | --- | --- | --- | --- |
|  |  |  |  | **Lower Bound** | **Upper Bound** |  |
| **Age group** | 18-30 | 31-43 | .37520 | .1604 | 1.9230 | .016 |
|  |  | 44 and more | .39709 | 1.0640 | 2.9294 | .000 |
|  | 31-43 | 18-30 | .37520 | -1.9230 | -.1604 | .016 |
|  |  | 44 and more | .45040 | -.1028 | 2.0129 | .086 |
|  | 44 and more | 18-30 | .39709 | -2.9294 | -1.0640 | .000 |
|  |  | 31-43 | .45040 | -2.0129 | .1028 | .086 |
| **Occupation status** | Housewife | University student | .57141 | -2.9230 | .4565 | .320 |
|  |  | Employed | .59038 | -1.8809 | 1.6108 | 1.000 |
|  |  | Retired | .86526 | -1.1868 | 3.9306 | .692 |
|  |  | Self-employed | .67298 | -1.3182 | 2.6620 | .954 |
|  |  | Laborer | 1.18068 | -2.7862 | 4.1967 | .997 |
|  |  | Unemployed | 1.39953 | -1.9667 | 6.3105 | .713 |
|  | University student | Housewife | .57141 | -.4565 | 2.9230 | .320 |
|  |  | Employed | .38169 | -.0305 | 2.2269 | .063 |
|  |  | Retired | .73880 | .4204 | 4.7898 | .008 |
|  |  | Self-employed | .50005 | .4264 | 3.3838 | .003 |
|  |  | Laborer | 1.09140 | -1.2890 | 5.1659 | .565 |
|  |  | Unemployed | 1.32507 | -.5133 | 7.3235 | .137 |
|  | Employed | Housewife | .59038 | -1.6108 | 1.8809 | 1.000 |
|  |  | University student | .38169 | -2.2269 | .0305 | .063 |
|  |  | Retired | .75356 | -.7215 | 3.7353 | .416 |
|  |  | Self-employed | .52161 | -.7356 | 2.3494 | .716 |
|  |  | Laborer | 1.10145 | -2.4169 | 4.0974 | .988 |
|  |  | Unemployed | 1.33336 | -1.6360 | 6.2499 | .596 |
|  | Retired | Housewife | .86526 | -3.9306 | 1.1868 | .692 |
|  |  | University student | .73880 | -4.7898 | -.4204 | .008 |
|  |  | Employed | .75356 | -3.7353 | .7215 | .416 |
|  |  | Self-employed | .81989 | -3.1245 | 1.7245 | .979 |
|  |  | Laborer | 1.27017 | -4.4227 | 3.0894 | .998 |
|  |  | Unemployed | 1.47580 | -3.5642 | 5.1642 | .998 |
|  | Self-employed | Housewife | .67298 | -2.6620 | 1.3182 | .954 |
|  |  | University student | .50005 | -3.3838 | -.4264 | .003 |
|  |  | Employed | .52161 | -2.3494 | .7356 | .716 |
|  |  | Retired | .81989 | -1.7245 | 3.1245 | .979 |
|  |  | Laborer | 1.14784 | -3.3610 | 3.4277 | 1.000 |
|  |  | Unemployed | 1.37194 | -2.5570 | 5.5570 | .930 |
|  | Laborer | Housewife | 1.18068 | -4.1967 | 2.7862 | .997 |
|  |  | University student | 1.09140 | -5.1659 | 1.2890 | .565 |
|  |  | Employed | 1.10145 | -4.0974 | 2.4169 | .988 |
|  |  | Retired | 1.27017 | -3.0894 | 4.4227 | .998 |
|  |  | Self-employed | 1.14784 | -3.4277 | 3.3610 | 1.000 |
|  |  | Unemployed | 1.68027 | -3.5022 | 6.4355 | .976 |
|  | Unemployed | Housewife | 1.39953 | -6.3105 | 1.9667 | .713 |
|  |  | University student | 1.32507 | -7.3235 | .5133 | .137 |
|  |  | Employed | 1.33336 | -6.2499 | 1.6360 | .596 |
|  |  | Retired | 1.47580 | -5.1642 | 3.5642 | .998 |
|  |  | Self-employed | 1.37194 | -5.5570 | 2.5570 | .930 |
|  |  | Laborer | 1.68027 | -6.4355 | 3.5022 | .976 |
| **Method of obtaining health information** | Physician/ Health care providers | Internet | .40562 | -1.9274 | .4715 | .552 |
|  |  | Newspapers/ magazines | 1.12142 | -3.4527 | 3.1797 | 1.000 |
|  |  | Friends and acquaintances | .68743 | -1.1235 | 2.9422 | .841 |
|  |  | Book | .69293 | -3.0196 | 1.0786 | .802 |
|  |  | Radio, television and satellite | .62969 | -.3035 | 3.4207 | .170 |
|  |  | I do not know | 1.00856 | -2.6137 | 3.3513 | 1.000 |
|  | Internet | Physician/ Health care providers | .40562 | -.4715 | 1.9274 | .552 |
|  |  | Newspapers/ magazines | 1.09117 | -2.6353 | 3.8182 | .998 |
|  |  | Friends and acquaintances | .63689 | -.2461 | 3.5207 | .137 |
|  |  | Book | .64282 | -2.1435 | 1.6583 | 1.000 |
|  |  | Radio, television and satellite | .57409 | .5889 | 3.9842 | .001 |
|  |  | I do not know | .97481 | -1.7860 | 3.9794 | .920 |
|  | Newspapers/ magazines | Physician/ Health care providers | 1.12142 | -3.1797 | 3.4527 | 1.000 |
|  |  | Internet | 1.09117 | -3.8182 | 2.6353 | .998 |
|  |  | Friends and acquaintances | 1.22421 | -2.5744 | 4.6660 | .979 |
|  |  | Book | 1.22730 | -4.4634 | 2.7953 | .994 |
|  |  | Radio, television and satellite | 1.19274 | -1.8321 | 5.2222 | .790 |
|  |  | I do not know | 1.42945 | -3.7218 | 4.7324 | 1.000 |
|  | Friends and acquaintances | Physician/ Health care providers | .68743 | -2.9422 | 1.1235 | .841 |
|  |  | Internet | .63689 | -3.5207 | .2461 | .137 |
|  |  | Newspapers/ magazines | 1.22421 | -4.6660 | 2.5744 | .979 |
|  |  | Book | .84927 | -4.3913 | .6315 | .289 |
|  |  | Radio, television and satellite | .79851 | -1.7121 | 3.0106 | .984 |
|  |  | I do not know | 1.12174 | -3.8577 | 2.7766 | .999 |
|  | Book | Physician/ Health care providers | .69293 | -1.0786 | 3.0196 | .802 |
|  |  | Internet | .64282 | -1.6583 | 2.1435 | 1.000 |
|  |  | Newspapers/ magazines | 1.22730 | -2.7953 | 4.4634 | .994 |
|  |  | Friends and acquaintances | .84927 | -.6315 | 4.3913 | .289 |
|  |  | Radio, television and satellite | .80325 | .1538 | 4.9045 | .028 |
|  |  | I do not know | 1.12512 | -1.9878 | 4.6665 | .898 |
|  | Radio, television and satellite | Physician/ Health care providers | .62969 | -3.4207 | .3035 | .170 |
|  |  | Internet | .57409 | -3.9842 | -.5889 | .001 |
|  |  | Newspapers/ magazines | 1.19274 | -5.2222 | 1.8321 | .790 |
|  |  | Friends and acquaintances | .79851 | -3.0106 | 1.7121 | .984 |
|  |  | Book | .80325 | -4.9045 | -.1538 | .028 |
|  |  | I do not know | 1.08731 | -4.4052 | 2.0255 | .930 |
|  | I do not know | Physician/ Health care providers | 1.00856 | -3.3513 | 2.6137 | 1.000 |
|  |  | Internet | .97481 | -3.9794 | 1.7860 | .920 |
|  |  | Newspapers/ magazines | 1.42945 | -4.7324 | 3.7218 | 1.000 |
|  |  | Friends and acquaintances | 1.12174 | -2.7766 | 3.8577 | .999 |
|  |  | Book | 1.12512 | -4.6665 | 1.9878 | .898 |
|  |  | Radio, television and satellite | 1.08731 | -2.0255 | 4.4052 | .930 |
| **Method of obtaining information related to mental illness** | Physician/ Health care providers | Psychologist/Psychiatrist | .86572 | -3.9103 | 1.2138 | .710 |
|  |  | Friends and acquaintances | .99343 | -4.5150 | 1.3651 | .692 |
|  |  | Book | .81644 | -4.8992 | -.0667 | .039 |
|  |  | Internet | .67458 | -4.0039 | -.0111 | .048 |
|  |  | Radio, television and satellite | 1.01628 | -4.4536 | 1.5617 | .789 |
|  |  | All Items above | .70932 | -4.3019 | -.1035 | .033 |
|  | Psychologist/Psychiatrist | Physician/ Health care providers | .86572 | -1.2138 | 3.9103 | .710 |
|  |  | Friends and acquaintances | 1.00126 | -3.1899 | 2.7365 | 1.000 |
|  |  | Book | .82595 | -3.5791 | 1.3096 | .816 |
|  |  | Internet | .68606 | -2.6896 | 1.3711 | .962 |
|  |  | Radio, television and satellite | 1.02393 | -3.1280 | 2.9326 | 1.000 |
|  |  | All Items above | .72024 | -2.9860 | 1.2771 | .899 |
|  | Friends and acquaintances | Physician/ Health care providers | .99343 | -1.3651 | 4.5150 | .692 |
|  |  | Psychologist/Psychiatrist | 1.00126 | -2.7365 | 3.1899 | 1.000 |
|  |  | Book | .95898 | -3.7461 | 1.9300 | .965 |
|  |  | Internet | .84149 | -2.9229 | 2.0578 | .999 |
|  |  | Radio, television and satellite | 1.13397 | -3.2270 | 3.4849 | 1.000 |
|  |  | All Items above | .86959 | -3.2013 | 1.9458 | .991 |
|  | Book | Physician/ Health care providers | .81644 | .0667 | 4.8992 | .039 |
|  |  | Psychologist/Psychiatrist | .82595 | -1.3096 | 3.5791 | .816 |
|  |  | Friends and acquaintances | .95898 | -1.9300 | 3.7461 | .965 |
|  |  | Internet | .62273 | -1.3675 | 2.3184 | .988 |
|  |  | Radio, TV | .98263 | -1.8710 | 3.9451 | .941 |
|  |  | All Items above | .66020 | -1.6735 | 2.2341 | 1.000 |
|  | Internet | Physician/ Health care providers | .67458 | .0111 | 4.0039 | .048 |
|  |  | Psychologist/Psychiatrist | .68606 | -1.3711 | 2.6896 | .962 |
|  |  | Friends and acquaintances | .84149 | -2.0578 | 2.9229 | .999 |
|  |  | Book | .62273 | -2.3184 | 1.3675 | .988 |
|  |  | Radio, television and satellite | .86835 | -2.0083 | 3.1314 | .995 |
|  |  | All Items above | .47365 | -1.5969 | 1.2066 | 1.000 |
|  | Radio, television and satellite | Physician/ Health care providers | 1.01628 | -1.5617 | 4.4536 | .789 |
|  |  | Psychologist/Psychiatrist | 1.02393 | -2.9326 | 3.1280 | 1.000 |
|  |  | Friends and acquaintances | 1.13397 | -3.4849 | 3.2270 | 1.000 |
|  |  | Book | .98263 | -3.9451 | 1.8710 | .941 |
|  |  | Internet | .86835 | -3.1314 | 2.0083 | .995 |
|  |  | All Items above | .89561 | -3.4073 | 1.8938 | .980 |
|  | All Items above | Physician/ Health care providers | .70932 | .1035 | 4.3019 | .033 |
|  |  | Psychologist/Psychiatrist | .72024 | -1.2771 | 2.9860 | .899 |
|  |  | Friends and acquaintances | .86959 | -1.9458 | 3.2013 | .991 |
|  |  | Book | .66020 | -2.2341 | 1.6735 | 1.000 |
|  |  | Internet | .47365 | -1.2066 | 1.5969 | 1.000 |
|  |  | Radio, television and satellite | .89561 | -1.8938 | 3.4073 | .980 |
